# A Fluorescent Real-Time Plaque Assay Enables Single-Cell Analysis of Virus-Induced Cytopathic Effect by Live-Cell Imaging

**DOI:** 10.3390/v13071193

**Published:** 2021-06-22

**Authors:** Jorge L. Arias-Arias, Eugenia Corrales-Aguilar, Rodrigo A. Mora-Rodríguez

**Affiliations:** 1 Centro de Investigación en Enfermedades Tropicales (CIET), Facultad de Microbiología, Universidad de Costa Rica, San José 11501-2060, Costa Rica; eugenia.corrales@ucr.ac.cr (E.C.-A.); rodrigo.morarodriguez@ucr.ac.cr (R.A.M.-R.); 2 Dulbecco Lab Studio, Residencial Lisboa 2G, Alajuela 20102, Costa Rica

**Keywords:** vesicular stomatitis, herpes simplex, yellow fever, animal viruses, plaque assay, real-time, live-cell imaging, automated image analysis, DNA fluorescent dyes, antiviral screening

## Abstract

Conventional plaque assays rely on the use of overlays to restrict viral infection allowing the formation of distinct foci that grow in time as the replication cycle continues leading to countable plaques that are visualized with standard techniques such as crystal violet, neutral red, or immunolabeling. This classical approach takes several days until large enough plaques can be visualized and counted with some variation due to subjectivity in plaque recognition. Since plaques are clonal lesions produced by virus-induced cytopathic effect, we applied DNA fluorescent dyes with differential cell permeability to visualize them by live-cell imaging. We could observe different stages of that cytopathic effect corresponding to an early wave of cells with chromatin-condensation followed by a wave of dead cells with membrane permeabilization within plaques generated by different animal viruses. This approach enables an automated plaque identification using image analysis to increase single plaque resolution compared to crystal violet counterstaining and allows its application to plaque tracking and plaque reduction assays to test compounds for both antiviral and cytotoxic activities. This fluorescent real-time plaque assay sums to those next-generation technologies by combining this robust classical method with modern fluorescence microscopy and image analysis approaches for future applications in virology.

## 1. Introduction

To date, plaque assay continues to be considered the “gold standard” virological technique for quantifying viral titers of lytic virions [1]. This method was originally described by d’Hérelle for the titration and isolation of bacteriophages [2] and later was modified and adapted by Dulbecco to animal viruses such as Western Equine Encephalitis virus and poliovirus [3,4]. The great advantage of plaque assay in viral titers determination lies in its capacity to quantify the current number of infectious viral particles within a sample [5].

The typical plaque assay relies on the use of solid or semisolid overlays (i.e., agarose or carboxymethyl cellulose, respectively) to restrict viral infection and spreading to the surrounding cells of the monolayer. This allows the formation of distinct foci of cell death that grow in time as the replication-lysis-infection cycle continues, finally arising as discrete and countable plaques that are visualized after fixation and counterstained with colorimetric dyes such as neutral red or crystal violet [5,6]. However, this classical approach takes several days until large enough plaques can be visualized and present wide variations in plaque recognition and counting among different analysts [1].

Alternatively, immunofluorescent staining of viral plaques have been used to increase the sensitivity and reduce the duration of plaque assays, since fluorescence labeling of early viral proteins enables faster identification of small plaques that are not visible by colorimetric counterstainings [1,7,8]. Nevertheless, this approach still requires cell fixation and a time-consuming immunolabeling procedure with virus-specific antibodies to reveal the plaques, making it expensive and not entirely amenable for high-throughput screenings.

Plaque growth implies viral replication, transcription, translation, release, and infection of surrounding cells [9]. Thus, the kinetic study of viral plaques formation in time at a cellular level could give valuable information about the infection established by a specific viral clone and can be used for the prediction of viral spreading behavior in vivo [9,10]. Such analysis requires live-cell imaging approaches for the kinetic visualization and monitoring of viral plaques on living cell monolayers, which presently can only be accomplished using transgenic fluorescent viruses constructed by extensive molecular work to modify the genome of reference strains [11,12,13].

Based on the fact that plaques are clonal lesions produced by virus-induced cytopathic effect, we applied DNA fluorescent dyes to label chromatin condensation and cell death as a way to visualize viral plaques by live-cell imaging. This approach allowed us to develop a fluorescent real-time plaque assay for the automated identification and tracking of individual viral plaques using a customized image analysis pipeline to monitor plaque formation kinetics at a single-cell level, using unlabeled wild-type viral strains. Such a procedure also enabled the automated characterization with a single-cell resolution of the cytopathic effect produced by both RNA and DNA animal viruses in terms of chromatin condensation and membrane permeabilization.

Furthermore, we describe the implementation of this novel approach for a real-time plaque reduction assay able to simultaneously screen both antiviral and cytotoxic effects in a single live cell imaging experiment. This approach will potentially enable a more efficient high-throughput screening of antiviral compounds and the evaluation of single-cell dynamics of infection within individual plaques to identify different clones within viral isolates.

## 2. Materials and Methods

### 2.1. Viruses

VSV-NJ Hazelhurst and HSV-1 F viruses were purchased from American Type Culture Collection (ATCC). Fluorescent HSV-1 F-ΔgE-GFP virus was kindly provided by David C. Johnson (Oregon Health and Science University) [11]. Vaccine strain YFV 17D was isolated in Vero cells from *Cercopithecus aethiops* (ATCC) using the commercial vaccine YF-VAX^®^ (Sanofi Pasteur) as inoculum. All viral stocks were produced in Vero cells by inoculating cellular monolayers at a multiplicity of infection (MOI) of 0.01–0.1 and incubating for 2–5 days with Minimum Essential Medium (MEM, Gibco) supplemented with 2% fetal bovine serum (FBS, Gibco) at 37 °C in an atmosphere of 5% CO_2_. Culture supernatants were collected, centrifuged at 3000× *g* for 10 min, aliquoted, and stored at −80 °C. Culture supernatant from uninfected Vero cells was also collected, stored, and used for mock infections. All viruses were titrated by plaque assay in Vero cells. Briefly, 10-fold serial dilutions of viruses were added to confluent monolayers of Vero cells. After 2 h of adsorption, cells were incubated at 37 °C in 5% CO_2_ for 5 days with MEM AutoMod™ (Sigma) supplemented with 2% FBS and 1% carboxymethylcellulose (Sigma). Plaque numbers were counted after staining with crystal violet. Virus inactivation was carried out by five cycles of UV light (254 nm) exposure at an energy of 400,000 J/cm^2^ in a CL-100 UV Cross-linker (UVP).

### 2.2. Live-Cell Imaging-Based Fluorescent Real-Time Plaque Assay

Vero cells were seeded on µClear black 96-well plates (Greiner Bio-One) at a density of 25,000 cells/well with MEM supplemented with 10% FBS. Wells from the periphery of the plate were not used to seed cells and were filled instead with 1X PBS in order to avoid desiccation in the wells with cells during long-term incubations. After 24 h of incubation at 37 °C with 5% CO_2_, cells were infected with 10-fold serial dilutions of either infectious or UV-inactivated VSV-NJ, HSV-1 F, HSV-1 F-ΔgE-GFP, or YFV 17D viruses. After 2 h of adsorption, cells were labeled with 1 µg/mL Hoechst 33342 (Invitrogen) for 10 min, washed once with 1X PBS supplemented with 1% FBS and incubated for 72–120 h at 37 °C −5% CO_2_ with MEM AutoMod^TM^ supplemented with 2% FBS, 1% carboxymethylcellulose, and 2.5 µg/mL of propidium iodide (Invitrogen) or 500 nM of SYTOX Green (Invitrogen). Washing and media addition steps were done fast and carefully in order to avoid desiccation that could kill and label with the death markers the cells on the periphery of the wells. The incubation was carried out using the above-mentioned conditions into the chamber of a Lionheart™ FX automated microscope (BioTek). Images of the whole well were acquired every 3–24 h. After the final read, viral plaques were confirmed by crystal violet staining. Automated plaque counts and single-cell analysis of viral plaques were performed by image analysis with the software CellProfiler 4.0 (http://www.cellprofiler.org; Broad Institute), using our previously reported pipelines for plaque identification (PlaqueIdentification.cpproj) and plaque tracking (PlaqueTracking.cpproj) [14], as well as a new customized pipeline for the quantification of chromatin-condensed and dead cells within individual plaques (PlaqueTrackingSX&Hoechst.cpproj, Supplementary Material). A detailed visual representation of this procedure is described in Figure S1.

### 2.3. Live-Cell Imaging-Based Fluorescent Real-Time Plaque Reduction Assay

Vero cells were seeded on a µClear black 96-well plate at a density of 25,000 cells/well with MEM supplemented with 10% FBS. After 24 h of incubation at 37 °C with 5% CO_2_, cells were infected with 10-fold serial dilutions of HSV-1 F. After 2 h of adsorption, cells were labeled with 1 µg/mL Hoechst 33342 for 10 min, washed once with 1X PBS supplemented with 1% FBS and incubated for 96 h at 37 °C-5% CO_2_ with MEM AutoMod^TM^ supplemented with 2% FBS, 1% carboxymethylcellulose, 2.5 µg/mL of propidium iodide, and different rising concentrations of acyclovir (Sigma, 0–3000 ng/mL). Images of the whole well were acquired at 96 h post-infection with a Lionheart™ FX automated microscope and viral plaques were confirmed by crystal violet staining. Automated viral plaques and dead cells counts were performed by image analysis with the software CellProfiler 4.0, using a customized pipeline called “Plaques&CytotoxicityAnalysis.cpproj” (Supplementary Material). The 50% inhibitory concentration (IC_50_), defined as the concentrations of antiviral required to reduce virus titers by 50%, as well as the 50% cytotoxic concentration (CC_50_), defined as the concentration of antiviral that reduces cell viability by 50%, were calculated using non-linear regression with the software GraphPad Prism 8.0. (GraphPad Software).

### 2.4. Statistics

Data are expressed as mean ± standard deviation (SD) of three independent experiments. Statistical significance of the differences between mean values was determined by using either an unpaired Student’s t-test or a one-way ANOVA followed by a Tukey’s post hoc test with the software GraphPad Prism 8.0. The level of significance is denoted in figure legends.

## 3. Results

### 3.1. The Differential Cell Permeability of DNA Fluorescent Dyes Enables the Visualization of Different Stages of the Cytopathic Effect at a Single-Cell Level within Individual Viral Plaques

DNA fluorescent dyes have differential cell permeability properties. Hoechst 33342 (referred hereafter as Hoechst) is typically used in fluorescence microscopy and flow cytometry to stain cell nuclei and chromatin condensation, an early marker of cell death since it is able to cross cell membranes of both living and dying cells [15,16]. In addition, cell-impermeant dyes such as SYTOX Green and propidium iodide (PI), are used to stain the nuclei of dead cells with terminal membrane permeabilization, which correlates with the final stages of a viral cytopathic effect [10,17]. To investigate whether we can translate those properties to observe a differential spatial distribution of the different stages of the virus-induced cytopathic effect, we implemented the staining of a bidimensional cell monolayer of infected viral cells with a late marker of cell death (SYTOX Green or PI) and an earlier marker for chromatin condensation (Hoechst).

**Figure 1:**
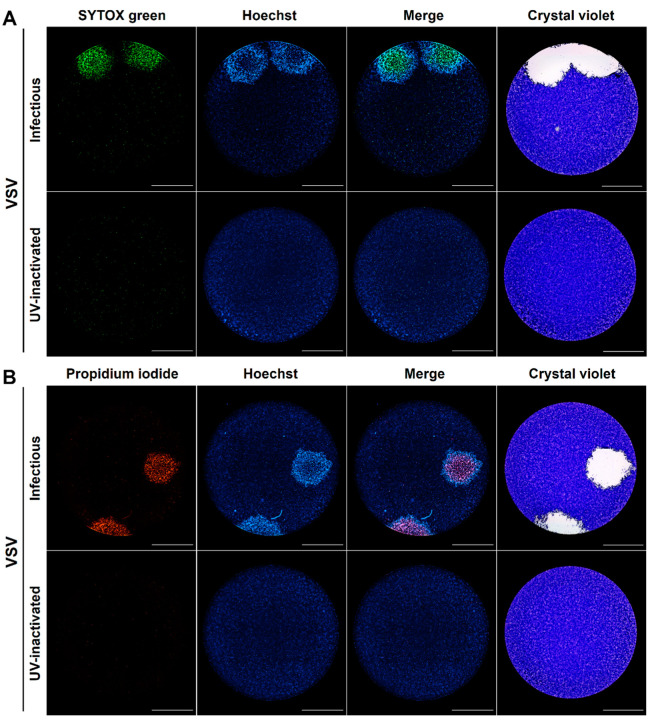
DNA fluorescent dyes with differential cell permeability enable the visualization by live-cell imaging of different stages of virus-induced cytopathic effect at a single-cell and single-plaque level. Vero cells were inoculated with either infectious or UV-inactivated VSV seeds, cell nuclei were stained with Hoechst, and cells were subjected to plaque assay for 72 h with a medium containing the cell death staining SYTOX Green (**A**) or propidium iodide (**B**). After live-cell imaging, acquisition viral plaques were confirmed by the standard crystal violet staining. A representative experiment is shown in each panel (n = three independent experiments), total magnification of 40X, scale bar = 2000 µm.

First, we assessed Vesicular Stomatitis Virus (VSV) plaque formation in Vero cells as a proof of principle with an RNA virus considered to have a lytic cytopathic effect [18]. Indeed, Hoechst labeled two larger plaques than those revealed by SYTOX Green. Interestingly, the staining with Hoechst was much weaker in the internal region of the plaque indicating a loss of chromatin staining in the zone stained by SYTOX Green. A merge of those two images shows that SYTOX Green-stained cells form an internal core of late cell death, suggesting that chromatin condensation occurs earlier than membrane permeabilization and this can be reflected as a differential spatial distribution (Figure 1A). A similar distribution was observed for VSV-infected PI stained cells together with Hoechst (Figure 1B). This differential distribution cannot be observed by crystal violet staining that includes both stages into a single cell-free counterstained area (Figure 1).

**Figure 2:**
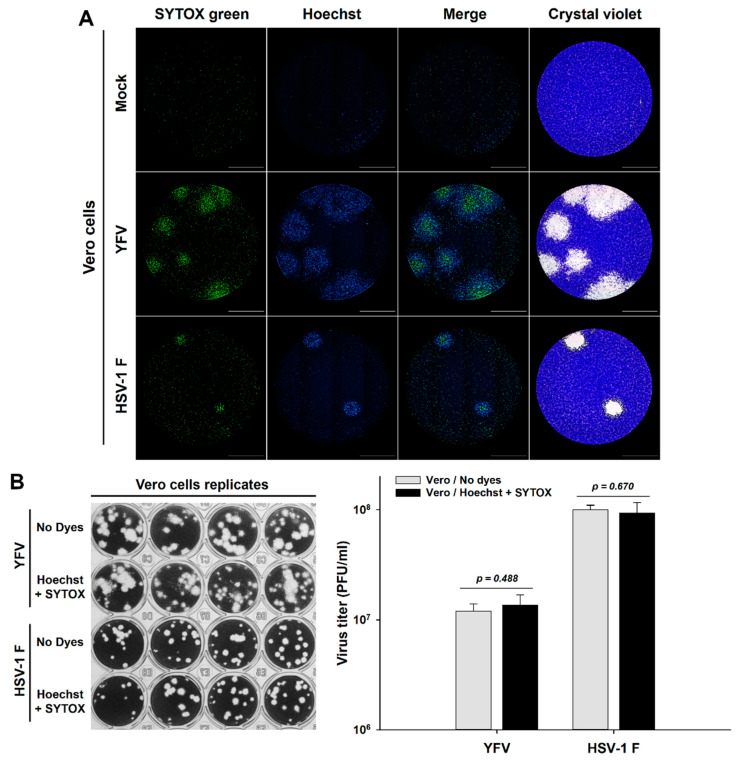
Labeling of virus-induced cytopathic effect with DNA fluorescent dyes has no effect on the replication of RNA and DNA animal viruses. (**A**) Vero cells were inoculated with either YFV or HSV-1 F seeds, cell nuclei were Hoechst stained, and cells were subjected to plaque assay during 120 h with a medium containing the cell death staining SYTOX Green. After live-cell imaging acquisition, viral plaques were confirmed by the standard crystal violet staining. (**B**) Vero cells were inoculated with either YFV or HSV-1 F seeds and subjected to plaque assay for 120 h both in the presence or absence of the DNA fluorescent dyes Hoechst and SYTOX Green. Plaques were revealed and counted using the standard crystal violet staining for the calculation of viral titers. A representative experiment is shown in each panel (n = three independent experiments), total magnification of 40X, scale bar = 2000 µm.

Second, we assessed the plaque formation of two viruses considered to have a non-cytolytic cytopathic effect, Yellow Fever Virus (YFV) and Herpes Simplex Virus (HSV) [9,19,20]. A similar spatial distribution was observed inside the plaques for both viruses with an inner core of membrane permeabilization (SYTOX Green) and an outer ring of chromatin condensation, which cannot be observed with crystal violet (Figure 2A). In addition, we assessed the effect of the current concentrations of those DNA fluorescent dyes on viral replication to rule out any interference in this assay. We revealed and counted the plaques using the standard crystal violet staining for the calculation of viral titers and observed no difference in viral replication in the presence or absence of these DNA dyes in Vero cells (Figure 2B). These results suggest that the staining with DNA fluorescent dyes with differential membrane permeability enables the visualization of different stages of the cytopathic effect at a single-cell level within individual viral plaques and indicates that DNA staining does not interfere with viral replication of both RNA and DNA animal viruses.

### 3.2. Time-Lapse Microscopy of Viral Plaques Labeled with DNA Fluorescent Dyes Enables the Real-Time Kinetic Identification of an Early Chromatin Condensation Wave Followed by a Membrane Permeabilization Wave with Single-Cell Resolution

**Figure 3:**
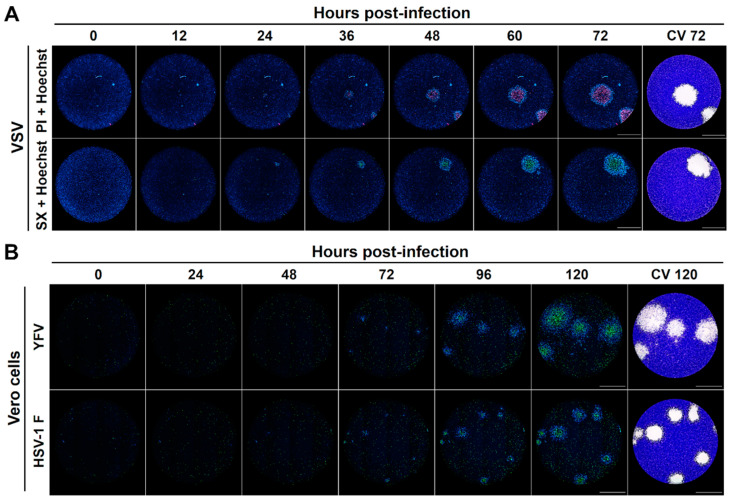
Labeling of individual viral plaques with DNA fluorescent dyes allows the development of a real-time plaque assay for the kinetic analysis of virus-induced cytopathic effect by live-cell imaging. (**A**) Vero cells were inoculated with infectious VSV seed, cell nuclei were stained with Hoechst, and cells were subjected to a kinetic plaque assay for 72 h with a medium containing one of the cell death dyes propidium iodide (PI) or SYTOX Green (SX). Live-cell imaging acquisition was performed every 12 h and after the final read viral plaques were confirmed by the standard crystal violet staining. (**B**) Vero cells were inoculated with either infectious YFV or HSV-1 F seeds, cell nuclei were Hoechst stained, and cells were subjected to a kinetic plaque assay for 120 h with a medium containing the cell death staining SYTOX Green. Live-cell imaging acquisition was performed every 24 h, and at the end of the experiment, viral plaques were confirmed by the standard crystal violet staining. A representative experiment is shown in each panel (n = three independent experiments), total magnification of 40X, scale bar = 2000 µm.

In order to ascertain whether the differential spatial distribution observed in the viral plaques stained with DNA fluorescent dyes correspond to time-resolved stages of the viral-induced cytopathic effect, we implemented a real-time plaque assay for the kinetic monitoring of viral plaques formation by live-cell imaging. VSV-infected Vero cells were monitored for 72 h after staining with Hoechst and in medium with either SYTOX Green or PI. Indeed, the first plaque-forming cells detected had an increased Hoechst staining and this behavior spread across the neighbor cells in the first wave of chromatin condensation that was followed by a second wave of plasma membrane permeabilization after a determined delay (Figure 3A, Video S1 and Video S2). Similar behavior was observed for YFV and HSV-1 F infection in Vero cells (Figure 3B).

**Figure 4:**
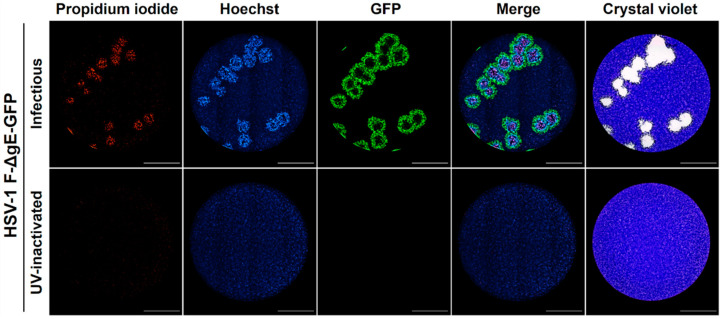
Comparison of DNA fluorescent dyes with a transgenic fluorescent virus for the identification of individual viral plaques by live-cell imaging. Vero cells were inoculated with either infectious or UV-inactivated HSV-1 F-ΔgE-GFP fluorescent virus, cell nuclei were Hoechst stained, and cells were subjected to plaque assay for 120 h with a medium containing the cell death dye propidium iodide. Viral plaques were confirmed by the standard crystal violet staining. Both fluorescent approaches allowed the identification of all plaques present in the samples analyzed. A representative experiment is shown (n = three independent experiments), total magnification of 40X, scale bar = 2000 µm.

To confirm these findings, we analyzed the viral plaques produced by a transgenic fluorescent HSV-1 (HSV-1 F-ΔgE-GFP) using live-cell imaging. This modified virus contains a GFP insert that confers green fluorescence to the infected cells [11]. Infected and Hoechst-stained Vero cells were subjected to plaque assay during 120 h with medium containing PI and then were analyzed by live-cell imaging for red (PI), blue (Hoechst), and green (GFP) fluorescence. Again, a differential spatial distribution was observed with small PI plaques that seem to be surrounded by larger Hoechst plaques and those by even larger GFP plaques. Both GFP and Hoechst images showed a decrease in fluorescence of their internal cores suggesting that a first wave of viable cells with viral replication (GFP) is followed by a wave of cells undergoing chromatin condensation (Hoechst) and finally by a wave of dead cells with membrane permeabilization (PI) (Figure 4). Those differences cannot be observed by classical crystal violet staining, confirming that the use of DNA fluorescent dyes with differential membrane permeability enables the time-resolved visualization of different stages of the virus-induced cytopathic effect at a single-cell level within individual viral plaques.

### 3.3. The Real-Time Plaque Assay with DNA Stains of Differential Membrane Permeability Enables Automated Identification of Single Viral Plaques with Higher Resolution When Compared to the Standard Crystal Violet Staining

**Figure 5:**
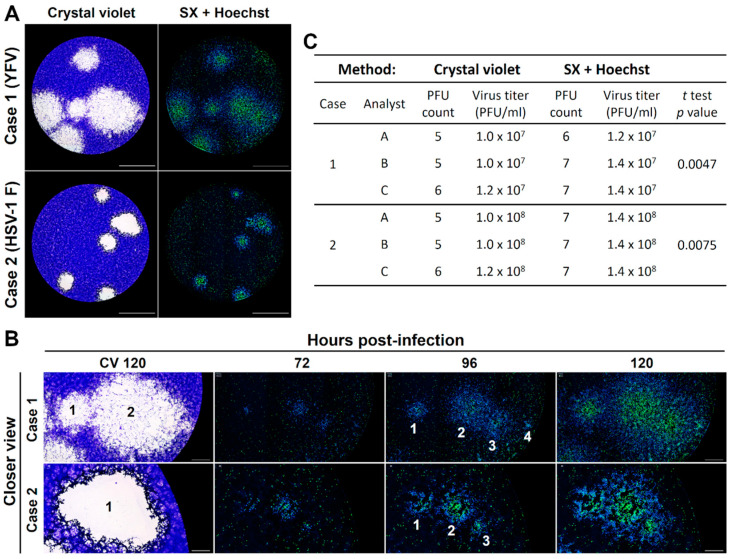
The fluorescent real-time plaque assay constitutes a better approach for the identification of single viral plaques when compared to the standard crystal violet staining. Two example cases of viral plaques generated by YFV (case 1) and HSV-1 F (case 2) in Vero cells were studied by both crystal violet staining and our real-time plaque assay with DNA fluorescent dyes (**A**). The kinetic analysis of viral plaques formation with our real-time plaque assay was a better approach for the calculation of the exact PFU count in those areas with plaque overlapping (**B**), as exposed by the differences in the PFU counts and final virus titers determined by three independent analysts using both the crystal violet staining and our real-time approach (**C**). Total magnification of 40X, scale bar = 2000 µm.

To evaluate the capability of a real-time plaque assay based on the staining of the different stages of viral cytopathic effect for individual plaque recognition and counting, we compared its performance to the standard crystal violet staining. We assessed two cases of viral plaques generated by YFV (case 1) and HSV-1 F (case 2) in Vero cells by those two different approaches upon interpretation by three different analysts (Figure 5A). For both cases, the plaque counts and calculated virus titers were significantly lower for the standard crystal violet staining compared to the plaques counted upon staining with Hoechst and SYTOX Green, due to an underestimation of the number of plaques in areas with plaque overlapping (Figure 5C). A closer view reveals that DNA staining increases the resolution in those areas, as it also allows the visualization of the development of individual plaques in time, enabling the identification of the initial focus of infection before overlapping (Figure 5B).

**Figure 6:**
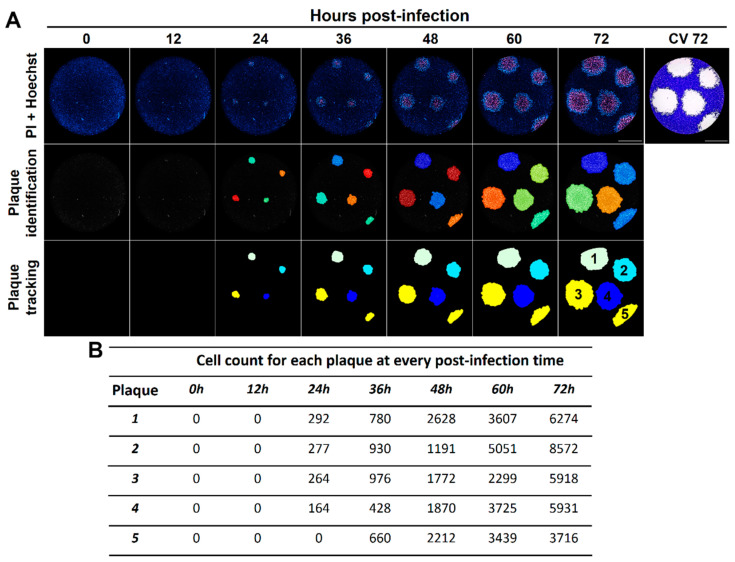
The fluorescent real-time plaque assay enables the automated analysis of the kinetics of viral plaques formation at a single-plaque and single-cell level. The kinetics of VSV plaques formation in Vero cells using our real-time plaque assay was analyzed with two different image analysis pipelines for plaque identification and plaque tracking (**A**), allowing the calculation of the cell counts for each identified viral plaque at every time point studied (**B**). A representative experiment is shown (n = three independent experiments), total magnification of 40X, scale bar = 2000 µm.

In order to automate the identification of viral plaques and their tracking over time, we analyzed images with our previously published image analysis pipelines developed in CellProfiler (PlaqueIdentification.cpproj and PlaqueTracking.cpproj) [14] obtaining a good time-resolved identification of individual plaques (Figure 6A). In addition, this approach allowed us to calculate the total number of cells composing the individual viral plaques over time (Figure 6B).

**Figure 7:**
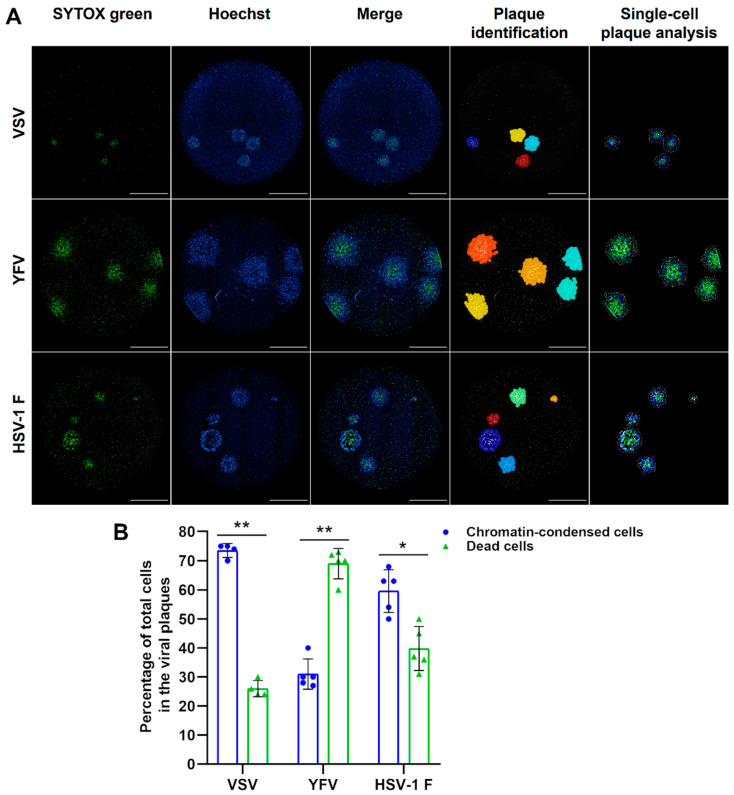
The fluorescent real-time plaque assay enables the automated comparative characterization of the cytopathic effect produced by different animal viruses in terms of chromatin condensation and induced cell death at a single-cell level. We applied an image analysis protocol to characterize the VSV, YFV, and HSV-1 F cytopathic effect by the quantification of fluorescence features of cells imaged to assess chromatin condensation (Hoechst) and cell death (SYTOX Green). (**A**) The image analysis protocol was applied to achieve the identification and single-cell analysis of the plaques produced by VSV at 36 h post-infection (hpi) and by YFV and HSV-1 F at 120 hpi, allowing the quantification and categorization of single cells within each plaque as chromatin-condensed (blue) and dead (green). (**B**) VSV, YFV, and HSV-1 F infection described by parameters of the percentage of chromatin-condensed and dead cells within individual viral plaques (blue dots and green triangles, respectively). Images and data from a representative analyzed experiment are shown (n = three independent experiments, magnification of 40X, scale bar = 2000 µm). Data are expressed as mean ± SD of the cell percentages calculated from the total number of plaques generated by each virus. * *p* < 0.001, ** *p* < 0.00001.

Furthermore, we developed a new image analysis pipeline (PlaqueTrackingSX&Hoechst.cpproj) to extract individual plaque information such as the total number of cells with chromatin condensation and plasma membrane permeabilization (death cells) for three animal viruses (VSV, YFV, and HSV-1 F, Figure 7A). With this approach, we noticed that the percentage of those cell subpopulations changes among different virus types (Figure 7B). These findings suggest that the ratio of single cells with a different stage of the viral cytopathic effect within a plaque could be used to differentiate plaque types in viral isolates from mixed infections or to identify mutant clones of the same virus strain. These results indicate that the labeling of viral plaques with DNA fluorescent dyes of differential cell permeability enables a high-resolution real-time plaque assay with the potential to be automated by the use of image analysis software to identify and characterize individual viral plaques.

### 3.4. The Combination of the Real-Time Plaque Assay with Automated Image Analysis Enables a High-Resolution Real-Time Plaque Reduction Assay for the Simultaneous Screening of Drugs in Terms of Antiviral and Cytotoxic Effect

Using classical virological methods for antiviral screening, a researcher must test in a first experiment the cytotoxicity of compounds to identify sublethal concentrations before attempting to test the antiviral activity in a subsequent experiment. To confirm the potential application of our real-time plaque assay to the screening of antiviral compounds, we aimed to develop a real-time plaque reduction assay that is able to simultaneously differentiate an antiviral effect from a cytotoxic effect. First, we inoculated Vero cells with infectious HSV-1 F virus, cell nuclei were stained with Hoechst and the cells were subjected to plaque assay for 96 h in the presence of medium containing PI and rising acyclovir concentrations ranging from 0 to 3000 ng/mL. Live-cell images were obtained at 96 h and the viral plaques were confirmed at the end of the experiment with crystal violet staining. Then, we aimed to develop a new advanced image analysis pipeline for the automated counting of viral plaques to test antiviral activities and the simultaneous measurement of dead cells present in uninfected zones outside viral plaques as a measure of the cytotoxicity induced by tested compounds.

**Figure 8:**
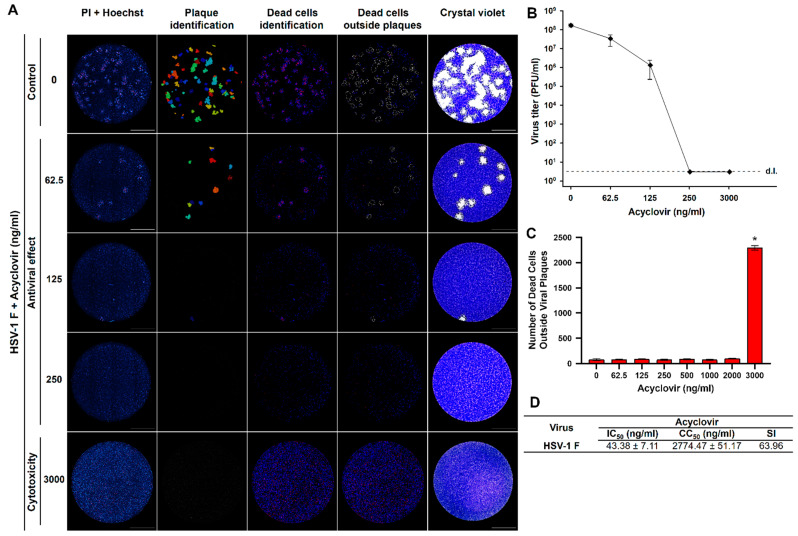
The fluorescent real-time plaque assay enables the application of a plaque reduction assay for the simultaneous screening of drugs in terms of antiviral and cytotoxic effects by live-cell imaging. (**A**) Vero cells were inoculated with infectious HSV-1 F virus, cell nuclei were Hoechst stained, and cells were subjected to plaque assay for 96 h with a medium containing the cell death staining propidium iodide (PI) and rising concentrations of acyclovir in the range 0–3000 ng/mL. After live-cell imaging acquisition, viral plaques were confirmed by the standard crystal violet staining. An image analysis pipeline was applied for the simultaneous counting and identification of viral plaques and the total number of dead cells. The plaque identification images were used to mask the dead cells identification images in order to obtain the number of dead cells outside the viral plaques, as a measure of the cytotoxicity generated by each concentration of the antiviral in non-infected cells. A representative experiment is shown (n = three independent experiments), total magnification of 40X, scale bar = 2000 µm. (**B**) The antiviral effect is exposed by the reduction in HSV-1 F titers produced by rising concentrations of acyclovir. d.l. = assay detection limit. (**C**) The cytotoxic effect of acyclovir is denoted by the statistically significant increment in the number of dead cells at the highest concentration tested (3000 ng/mL). * *p* < 0.001 compared to all other antiviral doses tested. (**D**) Calculated 50% inhibitory concentration (IC_50_), 50% cytotoxic concentration (CC_50_) and selectivity index (SI: CC_50_/IC_50_) for in vitro acyclovir treatment of HSV-1 F infection in Vero cells. Data are expressed as mean ± SD of three independent experiments.

Using such an image analysis pipeline (Plaques&CytotoxicityAnalysis.cpproj), we effectively quantified the number of HSV-1 F plaques to test the antiviral activity and the number of dead cells outside those plaques to simultaneously assess for cytotoxicity induced by acyclovir treatment (Figure 8A). This allowed us to demonstrate that the antiviral compound acyclovir indeed reduced the viral titer by several logarithms (Figure 8B) and that the concentration of 3000 ng/mL was cytotoxic, displaying a significant increase of dead cells compared to lower concentrations tested (Figure 8C). With these results, it was possible to calculate the 50% inhibitory concentration (IC_50_), 50% cytotoxic concentration (CC_50_), and selectivity index (SI: CC_50_/IC_50_) for in vitro acyclovir treatment of HSV-1 F infection in Vero cells (Figure 8D). These results demonstrate the potential application of these approaches for the automated and high-resolution image analysis of a real-time plaque reduction assay for the simultaneous screening of drugs in terms of antiviral and cytotoxic effects by live-cell imaging.

Taken together, our results indicate that the use of DNA fluorescent dyes with differential permeability enables the visualization and automated quantification of different stages of the cytopathic effect at a single-cell level. These stages correspond to an early chromatin condensation wave followed by a wave of dead cells with membrane permeabilization that can be observed by time-lapse microscopy with single-cell resolution within individual viral plaques. Moreover, this approach enables the implementation of fluorescent real-time plaque assays for the identification of single viral plaques with higher resolution when compared to the standard crystal violet staining and, if enhanced with advanced image analysis, it is possible to perform an automated real-time plaque reduction assay for the simultaneous screening of drugs in terms of both antiviral and cytotoxic effects by live-cell imaging.

## 4. Discussion

The present study reports the application of DNA fluorescent dyes with differential cell permeability for the development of a real-time plaque assay suitable for the automated single-cell analysis of the cytopathic effect induced by both RNA and DNA animal viruses within individual plaques. Such an approach represents a technological advance in the way that a plaque assay is conceived and expands the spectrum of utilities of this elegant virological technique that classically has been used mainly to determine infectious virus titers and for the isolation of individual viral clones [4,5,21].

However, plaques bear important information about the infection elicited by a particular virus at the cellular level. Since plaques are clonal lesions of infected cells formed by cell-to-cell or cell-free transmission of replicating viruses leading to a subsequent cytopathic effect [9,22,23], one can conceive plaques as models to study viral infection with an intrinsic type of timeline. Thereby, the first infected cells constitute the core of the plaques and newer neighboring and radial infections occur as the virus spreads to the periphery establishing a frontal wave of viral replication and infection. Moreover, the cell monolayer surrounding viral plaques is formed by non-infected healthy cells within the same well, constituting the perfect internal control of cell viability for the tested experimental conditions. Nevertheless, the classical counterstaining-based endpoint plaque assay using crystal violet does not leave cells for further analysis and even if plaques are revealed by immunofluorescence, such an approach still requires fixation hindering the investigation of the onset and kinetics of viral infection.

Therefore, we envisaged that DNA fluorescent dyes with differential cell membrane permeability could be used not only to label viral plaques but also to obtain important information about the virus-induced cytopathic effect at the cellular level, simultaneously enabling the analysis of infection kinetics in real-time by the live-cell imaging of the individual plaques. Using this rationale, we developed a fluorescent real-time plaque assay whereby we could observe a differential spatial distribution in the virus-induced cytopathic effect within viral plaques of VZV, YFV, and HSV-1 F, characterized by an early chromatin condensation wave arising first in the origin of the plaque and followed by a membrane permeabilization wave. This leads to a clear partition of the plaque with a core of terminally dead cells surrounded by a ring of cells with chromatin condensation in the periphery (Figure 1, Figure 2, Figure 3 and Figure 4, Video S1 and Video S2).

A deeper single-cell analysis of those time-resolved cellular subpopulations within viral plaques allowed us to identify interesting differences among virus types (Figure 7). We assessed the plaque formation by a virus considered to have a lytic cytopathic effect, VSV, and two other viruses considered to have a non-cytolytic cytopathic effect, YFV and HSV-1 F. Using our approach, we quantified the percentage of cells within each viral plaque corresponding to chromatin-condensed cells (Hoechst-intense cells) and dead cells with membrane permeabilization (PI or SYTOX Green positive cells). The observed variations indicate differential rates of infection and virus interaction with the cell death programs. The plaques produced by the infection with VSV have the highest proportion of chromatin condensed cells, suggesting that this VSV strain elicits a slow type of cell death in Vero cells, probably apoptosis as previously reported [24]. This finding suggests that VSV induces an early type of apoptosis reflected by a faster wave of chromatin condensation compared to the subsequent membrane permeabilization wave. Although this virus is typically considered highly cytolytic [18], Gadaleta and collaborators reported that VSV actually induces apoptosis at early stages in the viral cycle that does not depend on virus replication [24]. On the other hand, YFV-17D induces plaques with the highest proportion of dead cells suggesting a fast type of cell demise once the cell death program is engaged, probably necrosis [25]. Conversely, the cells with membrane permeabilization of the inner core of the YFV plaques keep also the Hoechst staining. This suggests that despite membrane permeabilization, the chromatin integrity is maintained, and, therefore, no empty space is visible within the Hoechst images of YFV plaques. Although YFV is typically considered as non-cytolytic [20], it was previously shown that different clones of YFV could induce small plaques or large plaques [21], probably reflecting different kinetics of cell death phases or a variable interaction with cell death mechanisms [26]. Indeed, it has been previously reported that YFV infection induces both pro-apoptotic [27] and anti-apoptotic [28] responses in vitro. Additionally, HSV-1 F has an intermediate behavior with a more similar proportion of chromatin-condensed and membrane-permeabilized cells, suggesting that HSV-1 F induced cell death has a relatively long phase of chromatin-related alterations before plasma membrane permeabilization, which is consistent with a budding virus able to delay late apoptosis and necroptosis [29]. Together, these results indicate that an intrinsic type of timeline of cell death-associated events is represented in the spatial distribution of chromatin-condensed and membrane-permeabilized cells within each individual viral plaque. These differences can potentially be used to distinguish viral clones or viral strains in mixed infections with potential applications to high-content screening assays of antiviral compounds affecting the rates of those cell death-associated events related to infection. Indeed, additional molecular sensors can be added to the cells in order to increase the content of information and the mechanistic insights that could be obtained about virus-induced alterations in a time-resolved manner. We have previously implemented such an approach with a reporter of flavivirus protease activity [10], but other interesting molecular sensors could be added to investigate the viral activities over different cellular processes like apoptosis [30] or autophagy [31], among others.

Yakimovich and collaborators [9] pointed out the utility of multi-parametric and automated kinetic analysis of viral plaques, however, they used transgenic viruses expressing fluorescent proteins to perform time-lapse microscopy, which up to date, is the method of choice to perform a kinetic viral plaque assay [12]. We reported an alternative approach based on the development of genetically modified reporter cell lines expressing a molecular sensor of viral infection [10]. Nevertheless, these methodologies are still virus-specific and extensive molecular biology work is required in order to develop and validate such modified viruses and cell lines. In contrast, the approach described in the present work is based on the use of low-cost DNA fluorescent dyes with differential cell membrane permeability, which not only allows the kinetic monitoring of plaque formation with unlabeled wild-type viral strains but also enables the characterization of the cytopathic effect produced by both RNA and DNA animal viruses in terms of chromatin condensation and induced cell death with single-cell resolution. In theory, this methodology could be applied to any plaque-forming virus as it involves the induction of cytopathic effect that could be labeled with the mentioned DNA fluorescent dyes.

The traditional plaque assay is multi-day, labor-intensive, and can be subjective due to visual inspection and manual plaque counting by different analysts. The time-resolved monitoring of viral plaques and the associated image analysis pipelines available here represent a technological advance compared to the classic crystal violet counterstaining. The approach presented here will pave the way towards an optimal identification and characterization of viral plaques with higher resolution, objectiveness, and information content. The image analysis pipelines (step-by-step protocols of image analysis) were developed in CellProfiler, an open-source software designed to share so that they could be available for all the interested scientific community (see Supplementary Material). We have developed a specific pipeline optimized to analyze the data of a real-time plaque reduction assay able to simultaneously screen for antiviral activities in plaques labeled with DNA dyes of differential membrane permeability and the cytotoxic effect of any chemical compound to be tested (Figure 8). Using this approach we were able to calculate an IC_50_ of 0.04 µg/mL of acyclovir for the inhibition of HSV-1 replication in vitro. These results are similar to those obtained with conventional plaque-reduction assays, as it has been previously reported that the IC_50_ of acyclovir against HSV-1 isolates ranges from 0.02 to 13.50 µg/mL [32,33,34,35]. This all-in-one assay will help to dissect confounding results of compounds with apparent antiviral activities that, in fact, lead to cellular alterations in viability or cell death subroutines that also impair viral replication by non-specific mechanisms.

Current advances in time-resolved microscopy and live-cell imaging are changing the methodological paradigm in many fields of biological sciences, including virology, which is reflected by the increasing number of procedures based on the kinetic visualization of the phenomenon being studied. Our fluorescent real-time plaque assay sums to those next-generation technologies by the combination of this robust classical method with the modern fluorescence microscopy and image analysis approaches. We envisaged that customized adaptations of this technology would be applied in future studies aimed to understand and decipher the mechanisms behind virus-host cell interactions, a pivotal knowledge for the development of vaccines and antiviral drugs to treat viral infections.

## Data Availability

Data is contained within the article.

## Supplementary Materials

The following are available online at https://www.mdpi.com/article/10.3390/v13071193/s1, Figure S1: Methodological workflow of the fluorescent real-time plaque assay, Video S1: Kinetics of VSV-NJ plaques formation in living Vero cells (0–72 h) using Hoechst 33342 and propidium iodide, Video S2: Kinetics of VSV-NJ plaques formation in living Vero cells (0–72 h) using Hoechst 33342 and SYTOX Green, CellProfiler image analysis pipelines: PlaqueIdentification.cpproj, PlaqueTracking.cpproj, PlaqueTrackingSX&Hoechst.cpproj, and Plaques&CytotoxicityAnalysis.cpproj.
