# Supplementary figures and images for: A Fluorescent Real-Time Plaque Assay Enables Single-Cell Analysis of Virus-Induced Cytopathic Effect by Live-Cell Imaging
Source: Viruses. 2021 Jun 22;13(7):1193. doi: 10.3390/v13071193 (PMC8310316; doi:10.3390/v13071193)

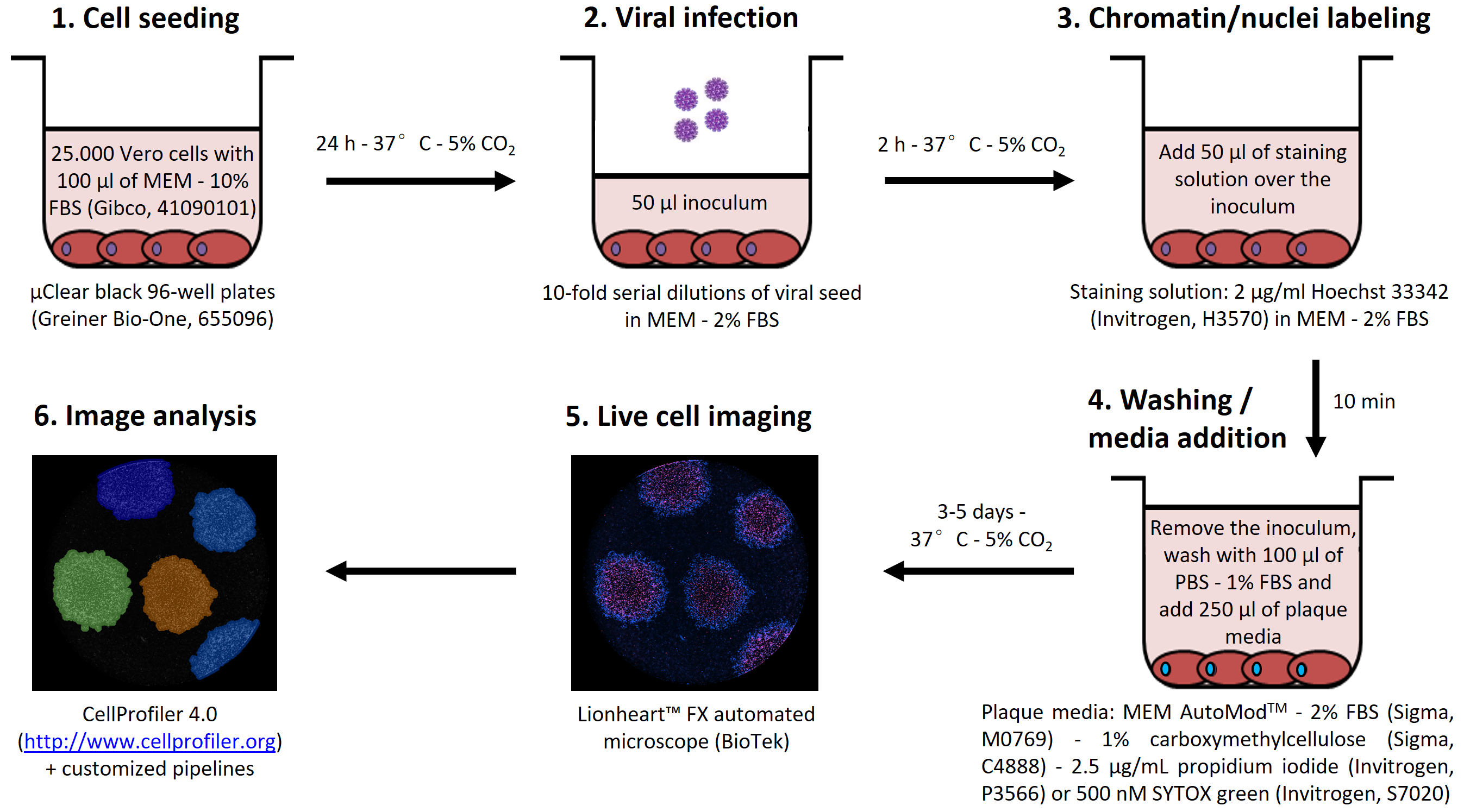

Supplement: Supplementary file 1 [file viruses-13-01193-s001.zip › Figure S1. Methodological workflow of the fluorescent real-time plaque assay.tif]
